# Assembly, annotation, and comparative analysis of Ipomoea chloroplast genomes provide insights into the parasitic characteristics of Cuscuta species

**DOI:** 10.3389/fpls.2022.1074697

**Published:** 2023-01-17

**Authors:** Yu Wang, Jing Xu, Bin Hu, Chunxing Dong, Jin Sun, Zixian Li, Kangzhuo Ye, Fang Deng, Lulu Wang, Mohammad Aslam, Wenliang Lv, Yuan Qin, Yan Cheng

**Affiliations:** ^1^ State Key Laboratory of Ecological Pest Control for Fujian and Taiwan Crops, College of Plant Protection, Fujian Agriculture and Forestry University, Fuzhou, China; ^2^ Provincial Key Laboratory of Haixia Applied Plant Systems Biology, Center for Genomics and Biotechnology, College of Agriculture, Fujian Agriculture and Forestry University, Fuzhou, China; ^3^ Clinical College of Chinese Medicine, Hubei University of Chinese Medicine, Wuhan, China; ^4^ College of Life Science, Fujian Agriculture and Forestry University, Fuzhou, China; ^5^ State Key Laboratory for Conservation and Utilization of Subtropical Agro-Bioresources, College of Agriculture, Guangxi University, Nanning, Guangxi, China; ^6^ Guangxi Key Lab of Sugarcane Biology, College of Agriculture, Guangxi University, Nanning, Guangxi, China; ^7^ Pingtan Institute of Science and Technology, Fujian Agriculture and Forestry University, Fuzhou, China

**Keywords:** chloroplast genome, Convolvulaceae, gene loss, parasitic plants, photosynthesis

## Abstract

In the *Convolvulaceae* family, around 1650 species belonging to 60 genera are widely distributed globally, mainly in the tropical and subtropical regions of America and Asia. Although a series of chloroplast genomes in *Convolvulaceae* were reported and investigated, the evolutionary and genetic relationships among the chloroplast genomes of the *Convolvulaceae* family have not been extensively elucidated till now. In this study, we first reported the complete chloroplast genome sequence of *Ipomoea pes-caprae*, a widely distributed coastal plant with medical values. The chloroplast genome of *I. pes-caprae* is 161667 bp in length, and the GC content is 37.56%. The chloroplastic DNA molecule of *I. pes-caprae* is a circular structure composed of LSC (large-single-copy), SSC (small-single-copy), and IR (inverted repeat) regions, with the size of the three regions being 88210 bp, 12117 bp, and 30670 bp, respectively. The chloroplast genome of *I. pes-caprae* contains 141 genes, and 35 SSRs are identified in the chloroplast genome. Our research results provide important genomic information for the molecular phylogeny of *I. pes-caprae*. The Phylogenetic analysis of 28 *Convolvulaceae* chloroplast genomes showed that the relationship of *I. pes-caprae* with *I. involucrata* or *I. obscura* was much closer than that with other *Convolvulaccae* species. Further comparative analyses between the *Ipomoea* species and *Cuscuta* species revealed the mechanism underlying the formation of parasitic characteristics of *Cuscuta* species from the perspective of the chloroplast genome.

## Introduction

*Ipomoea. pes-caprae*, which belongs to the *Convolvulaceae*, grows on coastal beaches and dunes throughout the tropical and subtropical areas of the world (Devall, 1992). This species has a high-speed growth rate and long-trailing stems, being one of the earliest species to colonize newly deposited dunes, contributing to the initial stabilization of sand (Devall and Thien, 1989). *I. pes-caprae* is widely distributed in southeast coastal areas of China, is found on tropical and subtropical beaches, and belongs to associated mangrove plants. It is common in Zhejiang, Fujian, Guangdong, Guangxi, and Taiwan. *I. pes-caprae* is evergreen all year round, with a particular leaf shape, strong growth potential, long flowering, and fruiting period. It has flowers almost all year round with bright colors. Its capsule is spherical, its pericarp is leathery, and its leaves, flowers, and fruits are of high ornamental value. The root system of *I. pes-caprae* is deeply grown into the soil to be used for sand fixation or covering plants on the beach. Recently, the medicinal value of *I. pes-caprae* has attracted the attention of researchers. It has been reported that the chemical components from *I. pes-caprae* have a wide range of biological activities for their antioxidant, analgesic and anti-inflammatory, antispasmodic, antinociceptive, antihistaminic, immunostimulant, insulinogenic, hypoglycemic antimicrobial, antifungal, and antibacterial characteristics (Bragadeeswaran et al., 2010). Moreover, previous studies have reported that *I. pes-caprae* could be used to inhibit platelet aggregation, diarrhea, vomiting, and piles (Manigauha et al., 2015).

Chloroplast is a vital organelle for green plants on earth to convert light energy into chemical energy (Jagendorf and Uribe, 1966; Neuhaus and Emes, 2000). The photosynthesis processes in the chloroplast are strictly regulated by a complex group of genes (Price et al., 2012). In plants, three organelles contain their genomes, nucleus, mitochondria, and chloroplast. The chloroplast genomes are highly conserved in genome structure, gene order, gene content, and gene number (Mira et al., 2018). Therefore, the chloroplast genomes were widely used as a valuable information resource for investigating the evolutionary history and taxonomic confirmation of land plants (Timme et al., 2007; Dong et al., 2014; Curci et al., 2015; Ellegren et al., 2015; Ju and Gao, 2016; Mira et al., 2018). The chloroplast genome is a circular double-stranded DNA molecule with a size of 120-180 KB, which is circular and consists of a large single-copy (LSC) region and a small single-copy (SSC) region separated by a pair of inverted repeats (Ozeki et al., 1989; Jansen et al., 2005; Petit et al., 2005; Funk et al., 2007; Jansen and Ruhlman, 2012). The chloroplast genome of land plants contains protein-coding genes and non-protein-coding genes. The protein-coding genes are mainly involved in photosynthesis and protein translation, and only a few are related to the transcription in the chloroplast. The non-protein-coding genes are the tRNA genes, whose transcripts are the transporters of amino acids in the peptide elongation, and the rRNA genes, composed of the ribosome (Jansen et al., 2005). The chloroplast genome can replicate by itself inside the chloroplast organelle. However, information communication and substance exchange with cytosol are critical for this biological event, and the genetic orders from the nuclei supervise all the metabolism in the chloroplast (Bulychev and Komarova, 2015).

In this study, the complete chloroplast genome sequence of *I. pes-caprae* was assembled, annotated, and comparatively analyzed. The results show that the length of the chloroplast genome is 161,667 bp with a GC content of 37.56%. The chloroplast genome of *I. pes-caprae* has a canonical structure, which is circular and composed of LSC, SSC, and IR regions, containing 136 annotated genes. The chloroplast genomes of 26 *Convolvulaceae* species, including 14 *Cuscuta* species and 12 *Ipomoea* species, were used for phylogenetic analysis and comparative analyses in codon preference and gene number, and gene content. Phylogenetic analysis showed that the relationship of *I. pes-caprae* with *I. involucrata* or *I. obscura* was much closer than that with other *Convolvulaccae* species. The phylogenetic and gene content analyses of *Convolvulaccae* species also provided new insight into the evolution of parasitic characteristics of *Cuscuta* species.

## Materials and methods

### Plant materials and DNA extraction

The *I. pes-caprae L*. plants used for this study were naturally growing on the beach located in Changle, Fuzhou (Latitude 25°, 54’, 33˝ N, Longitude 119°, 40’, 42˝ E), Fujian, China. The leaf materials were used for DNA extraction using the modified CTAB method (Li et al., 2013).

### Chloroplast genome assembly and annotation

The genome DNA samples were subject to SMART laboratory construction and then sequenced on the PacBio Sequel II sequencing platform. The CCS (Circular Consensus Sequence) reads corresponding to the chloroplast genome were extracted by mapping all the reads to the chloroplast genomes of all the *Convolvulaceae* species with Bowtie 2 (Langmead and Salzberg, 2012). Subsequently, the resulting CCS reads were considered to be derived from the chloroplast genome and were used for chloroplast genome assembly using Canu (V2.2) software (Nurk et al., 2020). The complete chloroplast genome sequences were annotated using the program PGA (Qu et al., 2019). Both tRNAs and rRNAs were identified by BLASTN and BLASTP by searching against the references composed of all the available chloroplast genomes of *Convolvulaceae* species. The annotation results were checked manually, and the codon positions were adjusted by comparing them to a previously homologous gene from various chloroplast genomes. The circular chloroplast genome map was drawn by the program Chloroplot (Zheng et al., 2020). The assembly and annotation of the chloroplast genome were submitted to NCBI (Accession No: MZ557416).

### Repeat sequence identification

Repeat elements in chloroplast genomes of *I. pes-caprae* were investigated using two different programs. The program MicroSAtellite identification tool (Katoh et al., 2002), (Beier et al., 2017) was used to identify the SSR repeat, setting the parameters with thresholds of 10, 5, 4, 3, 3, and 3 repeat units for mono-, di-, tri-, tetra-, penta-, and hexa-nucleotides, respectively. The program REPuter was used to detect the repeat sequences within the chloroplast genome (Kurtz et al., 2001). Four types of repeats, including forward repeats, reverse repeats, complement repeats, and palindromic repeats, were investigated in this analysis.

### Phylogenetic analysis

The chloroplast genome sequences of 26 *Convolvulaccae* species, including 14 *Cuscuta* species and 12 *Ipomoea* species, were used for phylogenetic analysis using three model species as outgroups (*Arabidopsis thaliana*, *Amborella trichopoda*, and *Oryza sativa*). The taxonomical details of the investigated species and the accession numbers of their chloroplast genome are listed in **Supplementary Table 1**. To accurately identify the phylogenetic position of *I. pes-caprae*, two phylogenetic trees were constructed based on the complete chloroplast genome sequences and the protein sequences, respectively. The alignment was conducted by MAFFT (Katoh et al., 2002), and the phylogenetic trees were constructed by MEGAX using Maximum Likelihood methods (Kumar et al., 2018).

### Comparative analysis of chloroplast genomes

To investigate the sequence divergence among *Ipomoea* species chloroplast genomes, The chloroplast genome of *I. pre-caprae* generated in this study, together with 11 released *Ipomoea* chloroplast genomes retrieved from NCBI, were used for comparative analysis. The sequences were aligned using the mVISTA program with Shuffle-LAGAN mode (https://pgrc.ipk-gatersleben.de/misa/) (Frazer et al., 2004).

### Identification of SNPs and hypervariable regions

To identify the SNPs and hypervariable regions within the chloroplast genome of *I. pes-caprae* in comparison with other *Ipomoea* species, the chloroplast genomes of other *Ipomoea* were aligned to the chloroplast genome of *I. pes-caprae* using MAFFT (Katoh and Standley, 2013). The nucleotide diversity (*Pi*) along the chloroplast genome was calculated using DnaSP version 5 software (Librado, 2009) with sliding window analysis. The window length was set to 800 base pairs, and the step size was set to 50 base pairs.

## Results

### Chloroplast genome assembly and annotation of *I. pes-caprae*

**Figure 1:**
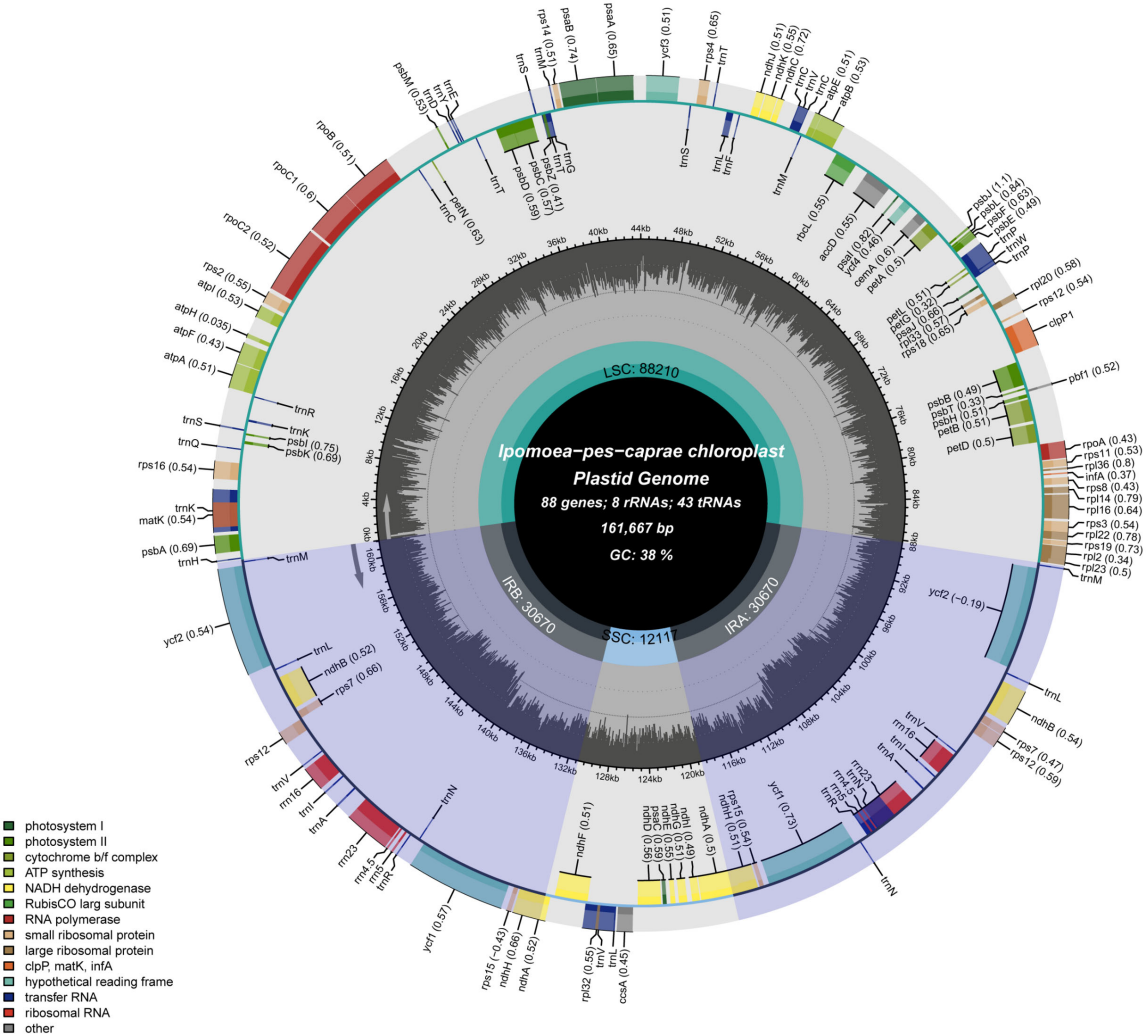
Circular gene map of *I. pes-caprae* chloroplast genome. Genes shown inside the circle are transcribed clockwise, and those outside are transcribed counterclockwise. Genes belonging to different functional groups are color-coded. The darker gray color in the inner circle corresponds to the GC content, and the lighter gray color corresponds to the AT content. LSC, SSC, and IR are large single-copy regions, small single-copy regions, and inverted repeat regions, respectively.

Using the PacBio Sequel II sequencing platform, 23252902 whole-genome long reads of *I. pes-caprae* were produced for genome assembly (unpublished project), and the mean read length is around 14 kb. Converted raw reads to CCS reads of the whole genome, the reads number from 23252902 to 1467275 (**Supplementary Table 2**). The chloroplast genome size of 161667 bp of *I. pes-caprae* was derived from the Canu program assembly. The *I. pes-caprae* complete chloroplast genome had a typical circular structure and with typical quadripartite organization consisting of the four conserved constitute regions, a pair of 30670 bp inverted repeats (IRs 30670 bp), an 88210 bp long single-copy regions (LSC), and a 12117 bp short single copy region (SSC) (**Figure 1**; **Supplementary Table 3**).

A number of 136 functional genes were identified in the chloroplast genome of *I. pes-caprae*, including 86 protein-coding genes, 42 tRNA genes, and 8 rRNA genes (16S, 23S, 5S, and 4.5S) (**Figure 1**; **Table 1**). According to their functions, the 136 *I. pes-caprae* chloroplast genes were assigned into three categories: Most protein-coding genes are related to photosynthesis. There are 45 genes in this category, including genes encoding subunits of ATP synthase, subunits of photosystem II, subunits of cytochrome b/f complex, subunits of NADH-dehydrogenase, assembly/stability of photosystem I, subunits of photosystem I, subunits of photosystem II, cytochrome c synthesis (*ccsA*), photosystem biogenesis factor 1 (*pbf1*), and subunit of rubisco (*rbcL*). The second category consists of 86 genes associated with chloroplast transcription and translation, including protein-coding genes encoding the large subunits of the ribosome, the small subunits of ribosomal proteins, DNA-dependent RNA polymerase, and translational initiation factor, and two types of non-coding genes, i.e., rRNA genes, tRNA genes. And the rest of the four genes were classified as other genes because of their unique or unknown function, including *matK* with function in RNA processing and 3 conserved open reading frames (*ycf1* and *ycf2*×2) encoding proteins of unknown function (**Table 1**; **Supplementary File 3**).

**Table 1:** The annotated genes in the chloroplast genome of I. pes-caprae.

Category for genes	Group of gene	Name of gene
**Photosynthesis**	Subunits of ATP synthase	*atpA, atpB, atpE ,atpF, atpH, atpI*
	Subunits of photosystem II	*psbE, psbF*
	Subunits of cytochrome b/f complex	*petA,petB,petG,petD,petL,petN*
	Subunits of NADH-dehydrogenase	*ndhA (×2),ndhB (×2), ndhC, ndhD, ndhE, ndhF, ndhG, ndhH (×2), ndhI, ndhJ, ndhK*
	Assembly/stability of photosystem I	*ycf3, ycf4*
	Subunits of photosystem I	*psaA, psaB, psaC, psaI, psaJ*
	Subunits of photosystem II	*psbA, psbB, psbC, psbD, psbH, psbI, psbJ, psbK, psbL, psbM, psbT, psbZ*
	cytochrome c synthesis	*ccsA*
	photosystem biogenesis factor 1	*pbf1*
	Subunit of rubisco	*rbcL*
**Transcription and translation**	Large subunits of ribosome	*rpl14, rpl16, rpl2, rpl20, rpl22, rpl23, rpl32, rpl33, rpl36*
	Small subunits of ribosomal proteins	*rps11, rps12 (×2), rps14, rps15 (×2), rps16, rps18, rps19, rps2, rps3, rps4, rps7 (×2), rps8*
	DNA dependent RNA polymerase	*rpoA, rpoB, rpoC1 (×2), rpoC2*
	Translational initiation factor	*infA*
	rRNA genes	*rrn16 (×2), rrn23 (×2), rrn4.5, rrn4.5, rrn5 (×2)*
	tRNA genes	*trnfM-CAU, trnI-CAU, trnI-CAU, trnA-UGC (×2), trnC-ACA, trnC-GCA, trnD-GUC, trnE-UUC, trnF-GAA, trnG-GCC, trnH-GUG, trnI-GAU (×2), trnK-UUU (×2), trnL-CAA (×2), trnL-UAA, trnL-UAG, trnM-CAU (×4), trnN-GUU (×3), trnP-UGG (×2), trnQ-UUG, trnR-ACG (×2), trnR-UCU, trnS-GCU, trnS-GGA, trnS-UGA, trnT-GGU (×2), trnT-UGU, trnV-AAC, trnV-GAC (×2), trnV-UAC, trnW-CCA, trnY-GUA*
**Other genes**	maturase K	*matK*
	Conserved open reading frames	*ycf1,ycf2 (×2)*

Genes with multiple copies were marked with (×2, ×3), indicating the genes had two or three copies.

### The sequence repeats in *I. pes-caprae* chloroplast genome

The sequence repeats widely exist in eukaryotic genomes, and the simple sequence repeat (SSR) is the most abundant and typical repeat type. SSRs in chloroplast genomes exhibit high copy numbers, which play an essential role in genome rearrangement and recombination and are important molecular markers in plant phylogenetic and evolutionary studies (Kuang et al., 2011; Yang et al., 2011; Jiao et al., 2012; Xue et al., 2012). From the perspective of evolution, the differences in repetitive sequences among species resulted from natural selection (Huang et al., 2021). 35 SSRs were identified in the chloroplast genome of *I. pes-caprae* (**Supplementary Table 4**): 32 are A/T single nucleotide repeats, and one is the TA dinucleotide repeat. Notably, there was no di, tri-, tetra-, penta-, or hexanucleotide repeat detected in the chloroplast genome of *I. pes-caprae*. The longer sequence repeats within the chloroplast genome were identified using Reputer 2.0 software. As a result, 50 repeats consisting of 26 forward and 24 palindromic repeats were obtained, while no complement or reverse repeats were detected (**Supplementary Table 5**). The largest repeat unit with a size of 30670 bp is the inverted repeats of chloroplast, which is essential for the chloroplast structure organization. And the lengths of the rest of the repeat units ranged from 119 and 242 bp. The repeat information of the chloroplast genome of *I. pes-caprae* is valuable for developing genetic markers for phylogenetic and population studies (Nie et al., 2012).

### Codon usage patterns in *Ipomoea* chloroplast genomes

**Figure 2:**
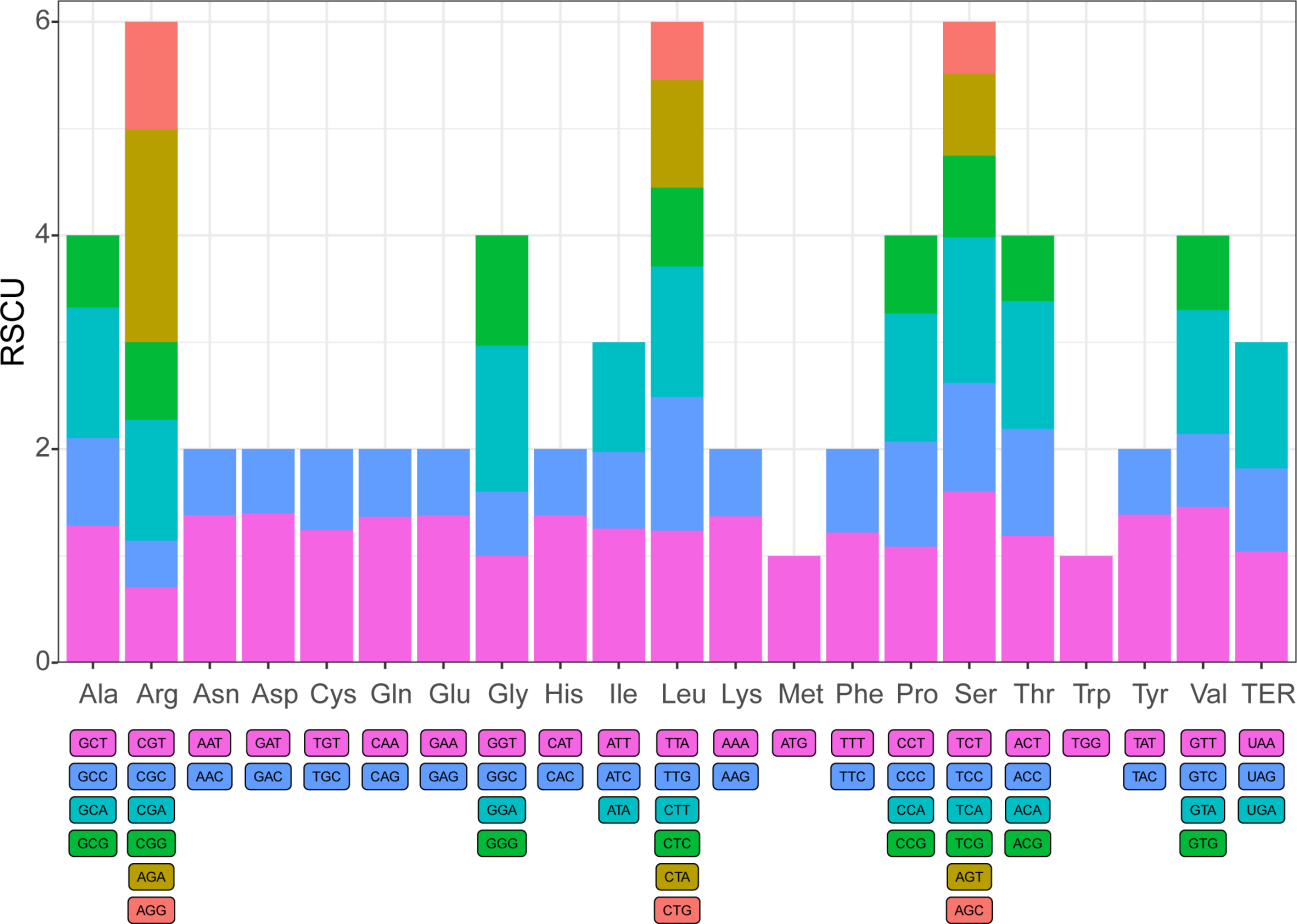
The Condon preference of the chloroplast genome of *I. pes-caprae*. Codon content of 20 amine acid and stop codons in all protein-coding genes of the chloroplast genome of *I. pes-caprae*.

In evolution, species are affected by natural selection pressure and genetic drift, resulting in differences in the use frequency of most genetic codons. Thus, the different genomes might have specific codon preferences (Hershberg and Petrov, 2008). In the complete chloroplast genome of *I. pes-caprae*, There are 53889 codons within the protein-coding genes. The *I. pes-caprae* chloroplast genome encoded all 20 amino acids, and 61 types of amino-acid codons were observed (**Supplementary Table 6**). The UU-started codons are found to be more frequent than the others (**Supplementary Table 7**). Among the 20 amino acids, Leucine was the most abundant (number of codons encoding Leucine = 5708, 10.59%), Serine was the second abundant (number of codons encoding Serine = 4961, 9.21%), while the rarest one is Tryptophan (713 codons, approximately 2.12%). Thirty-three codons were observed to be used more frequently than the expected usage at equilibrium (RSCU (Relative Synonymous Codon Usage) > 1), and 27 codons showed the codon usage bias (RSCU < 1). However, the frequency of use for the AUG (Methionine, start codon), UGG (Tryptophan), ACC (Threonine), and GGU (Glycine) showed no bias (RSCU = 1). In aspects of amino acids, most of them have codon preferences, except for Methionine and Tryptophan (**Figure 2**). And most of the 20 amino acids with at least two codons, and the acids Arginine, Leucine, and Serine have six codons. To investigate whether the codon usage preferences are conserved among the species in the *Ipomoea* genus, the protein-coding genes of the other 11 *Ipomoea* species, including *I. batatas* (53822 amino acids), *I. hederifolia* (53791), *I. involucrata* (53506), *I. minutiflora* (53729), *I. murucoides* (53327), *I. nil* (53965), *I. obscura* (53749), *I. purpurea* (54015), *I. tricolor* (53589*)*, *I. trifida* (53711), and *I. triloba* (53916) were investigated. As shown in **Supplementary File 1**, the codon usage preferences of all the 11 investigated species showed a similar tendency as that of *I. pes-caprae* (**Supplementary File 1**), indicating the conservation of codon bias in the genus of *Ipomoea*, which might be because that the ancestors of those species underwent the shared evolutionary history.

**Figure 3:**
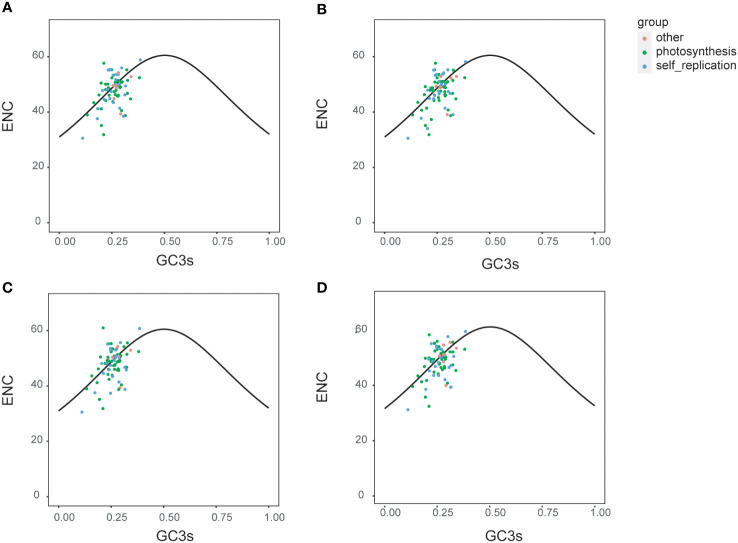
Enc plotted against GC3s of three *Ipomoea* species. The solid lines represented the expected curve of positions of genes when the codon usage was only determined by the GC3s composition. Enc and GC3s plots for four *Ipomoea* species, including *I pes-caprae* **(A)**, *I involucrate* **(B)**, *I murucoides* **(C)**, and *I tricolor* **(D)**.

The ENc plots are usually used to indicate the factors that affect the codon bias. To understand the relative importance of natural mutation and evolutionary selection in producing codon usage patterns, ENc (Effective Number of Codons) values of four *Ipomoea* species (*I. pes-caprae*, *I. involucrata*, *I. murucoide*, and *I. tricolor*) were estimated and plotted against the GC3s values (**Figure 3**). From the ENc plots, it is clear that the protein-coding genes of the four species showed similar codon bias patterns. Most of the genes were distributed on both sides of the standard curve, and more than half of the genes were below the curve, suggesting that the selection pressures predominantly influence codon bias in the chloroplast genome of the *Ipomoea* species. Photosynthesis-related genes are distributed most discretely, suggesting that some other factors might influence the codon bias, or maybe these genes are more conserved than self-replication and other group genes.

### Expansion and contraction of the IR regions

**Figure 4:**
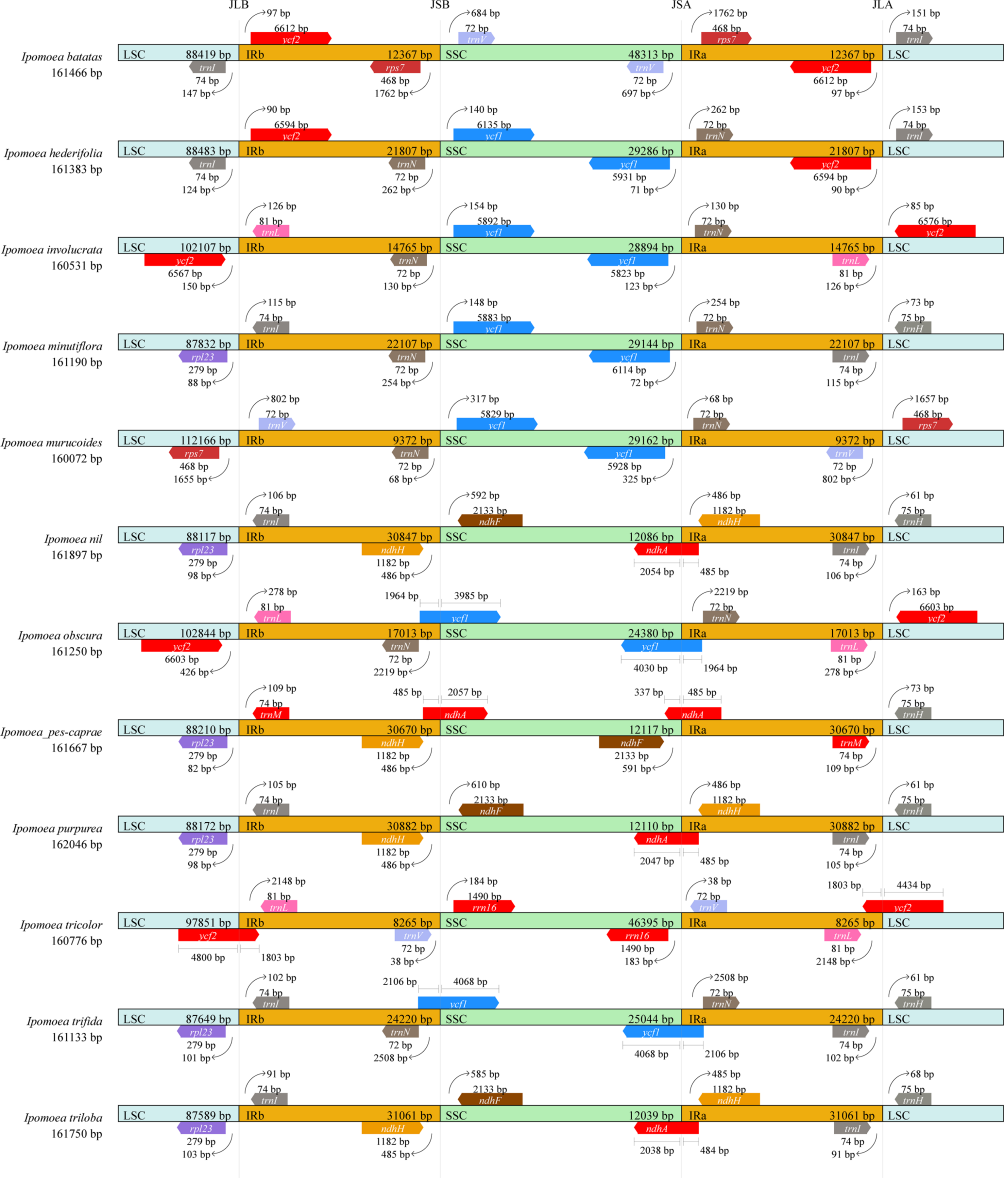
Comparison of the borders of the IR, SSC, and LSC regions among 12 chloroplast genomes of *Ipomoea species*. JLB, JSB, JSA, and JLA represent the junctions of LSC/IRb, IRb/SSC, SSC/IRa, and IRa/LSC, respectively.

Expansion and contraction of IR regions are common events that frequently happen in the evolutionary history of land plants (Raubeson et al., 2007; Kode et al., 2010; Yao, 2015). LSC/IR and IR/SSC junctions are sometimes regarded as an index of chloroplast genome evolution. The map illustrating the LSC/IRb (JLB), IRb/SSC (JSB), SSC/IRa (JSA), and IRa/LSC (JLA) boundaries/junctions of the 12 *Ipomoea* chloroplast genomes was constructed according to heir chloroplast genome sequences and annotation information (**Figure 4**). It is shown that the chloroplast genome organizations are highly conserved across the 12 *Ipomoea* species with only minor variations. The chloroplast genomes of 12 species have similar sizes ranging from 160072 bp (*I. murucoides)* to 161897 bp (*I. nil*). However, the sizes of LSC, SSC, and IR are varied significantly. For example, The lengths of IRs ranged from 8265 bp (*I. tricolor*) to 31061 bp (*I. triloba*), and the size of IR of *I. pes-caprae* was 30670 bp. The LSC/IR and SSC/IR boundaries in all 12 *Ipomoea* species were distributed with different genes, and only a few chloroplast genomes with consensus genes. The RS lines (the boundary lines between IRb/IRb and SSC) were mainly located between *ycf1* and *trnN*.

On the contrary, the RL lines (the boundary lines between IRa/IRb and LSC) were more variable than RS lines. Compared to species of other genera, the LSC regions of *I. involucrata*, *I. murucoides*, *I. obscura*, and *I. tricolor* have the expansion of *ycf2* to the IR LSC region, decreasing IR length. On the other hand, the SSC Regions of *I. pes-caprae*, *I. nil*, *I. purpurea*, and *I. triloba* were concentrated compared to those of other species, with the loss of *ycf1* in the SSC regions. As previous studies suggested, the expansion and contraction detected in the IR regions might be a primary mechanism in the length variation of three regions (LSC, SSC, and IR) of the chloroplast genomes in *Ipomoea* species (Sun et al., 2019).

### Phylogenetic analysis of chloroplast genomes of *Convolvulaceae* plants

**Figure 5:**
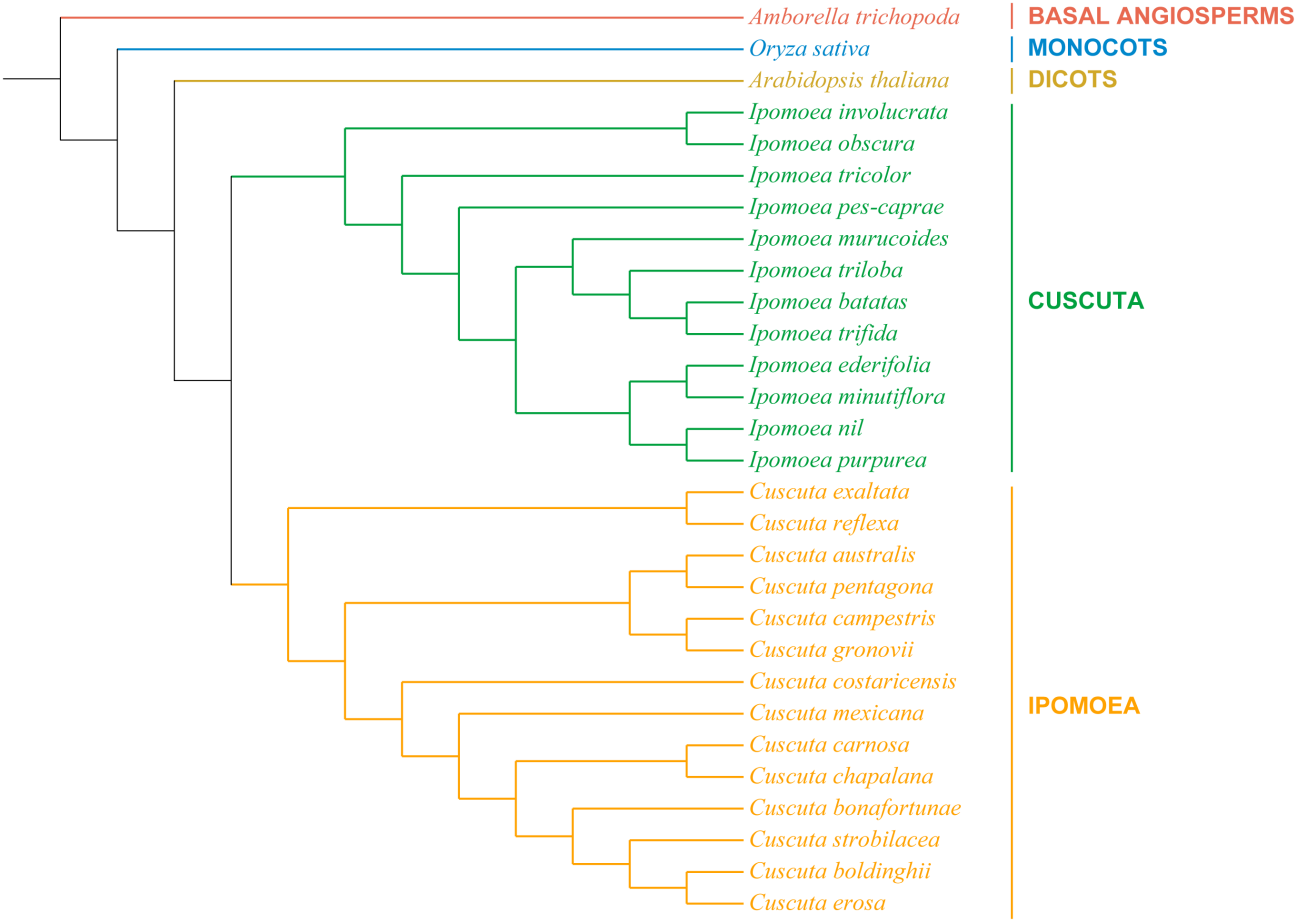
The phylogenetic relationships of the 26 *Convolvulaceae* species. Orthologous Single-copy genes phylogenetic tree of 26 *Convolvulaceae* family species and three outgroups constructed using maximum likelihood (ML) algorithm.

In order to understand the evolutionary relationships of *I. pes-caprae* with the *Ipomoea* genus, the chloroplast genomes of 28 *Convolvulaccae* species and three outgroup species (*A. thaliana*, *A. trichopoda*, and *O. sativa*) were used for phylogenetic analysis. The complete chloroplast genomes were downloaded from NCBI (National Center for Biotechnology Information database) and used for constructing the phylogenetic trees. We first constructed the phylogenetic trees based on the complete chloroplast genome sequences (**Figure S1**). The *Convolvulaccae* species were classified into the *Ipomoea* clade and the *Cuscuta* clade. The phylogenetic tree generated based on completed chloroplast sequences showed consistent relationships with taxonomical classification, suggesting confidence in the phylogenetic analysis based on the chloroplast genome. However, one of the outgroups species, *A. trichopoda*, the base angiosperm, was not in the basal position in the phylogenetic tree. One reason might be because of the sequence variation of the non-coding sequences. To this end, the sequences of orthologous single-copy genes were used to construct a maximum likelihood tree. As shown in **Figure 5**, the Basal Angiosperms, Monnocotes, and Diocts outgroups species showed a clear taxonomical relationship, and the 12 *ipomoea* species and 14 *Cuscuta* species were clustered into two clades. Our results showed that the chloroplast genomes could be used for constructing the phylogenetic tree reflecting the evolutionary relationships of land plants, and the orthologous single-copy genes sequence-based phylogenetic relationships based would be much more confident than the complete chloroplast genome sequences-based phylogenetic relationships.

### Comparative analysis between *Ipomoea* and *Cuscuta* species

**Figure 6:**
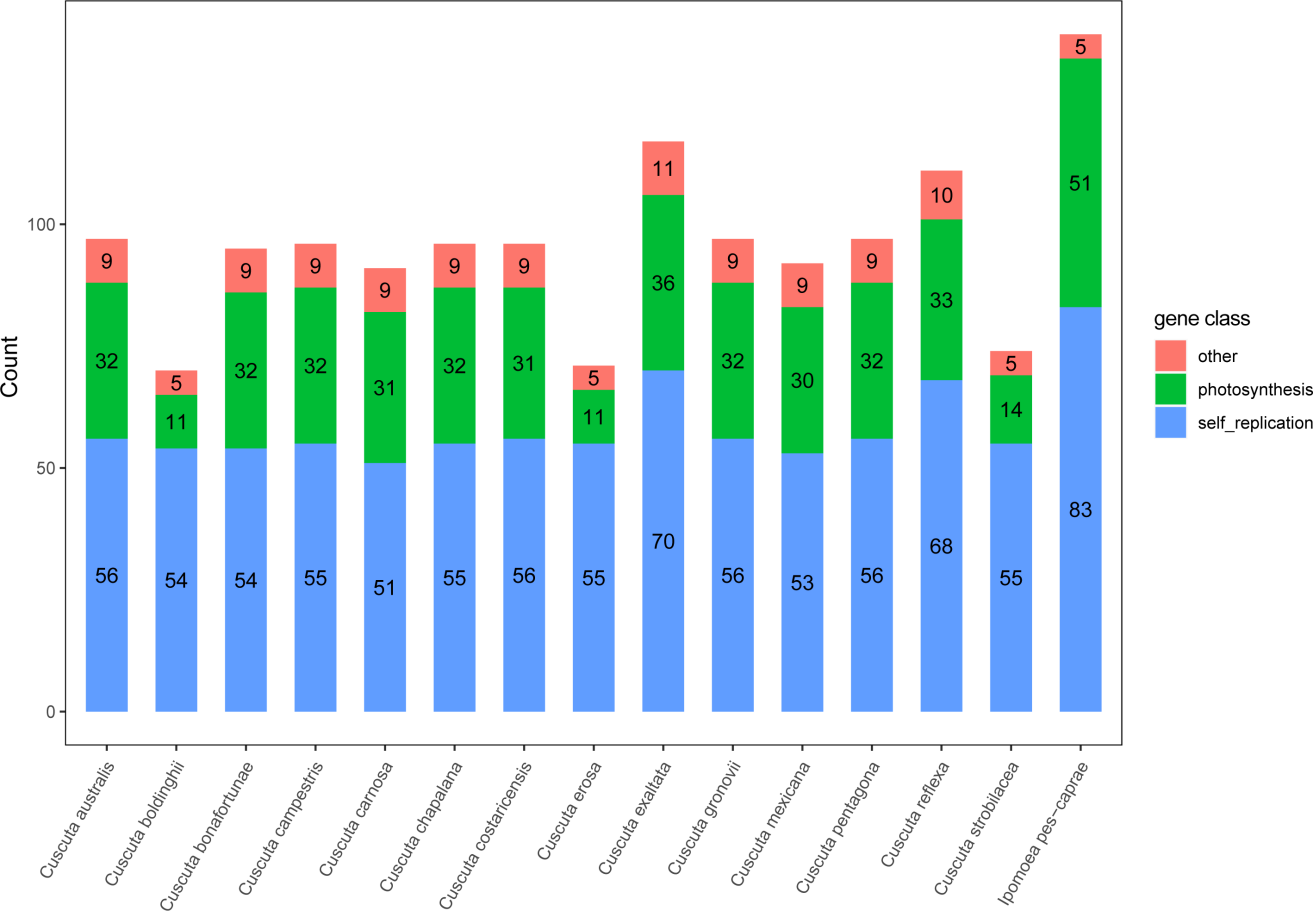
The number of three types of genes in the chloroplast genome of *Cuscuta* species. Red represents genes with other functions, green represents photosynthesis-related genes, and blue indicates genes involved in the self-replication of the chloroplast genome.

*Cuscuta spp* plants are annual parasitic herbs, and most of them are leafless, lack chlorophyll, and have a wide range of hosts (Albert et al., 2008). By comparing the protein-coding genes between *Cuscuta* and *Ipomoea* species, it’s evident that many genes were lost in *Cuscuta* species (**Supplementary Table 8**). In *Cuscuta* species, only *C. exaltata* maintains a few *ndh* (Subunits of NADH-dehydrogenase) genes. The other 13 *Cuscuta* species missed all of the *ndh* genes related to the photosynthesis pathway (**Figure 6**; **Supplementary File 2**). The result of the comparative analysis showed that *C. boldinghii*, *C. erosa*, and *C. strobilacea* have the least number of genes, which are 31, 33, and 33, respectively, and *C. exaltata* has the most number of genes, which is 67. Except for *C. exaltata*, other *Cuscuta* species don’t have complete photosynthesis-related genes, such as the genes coding subunits of cytochrome b/f complex, subunits of photosystem I, and subunits of photosystem II. Therefore, it is concluded that the loss of genes involved in photosynthesis in *Cuscuta* chloroplast genomes happened gradually, and *Cuscuta* species lost the photosynthesis ability to various extents, which is consistent with their nutritional performance.

### Chloroplast genome comparative analysis between *I. pes-caprae* and other *Ipomoea* species

**Figure 7:**
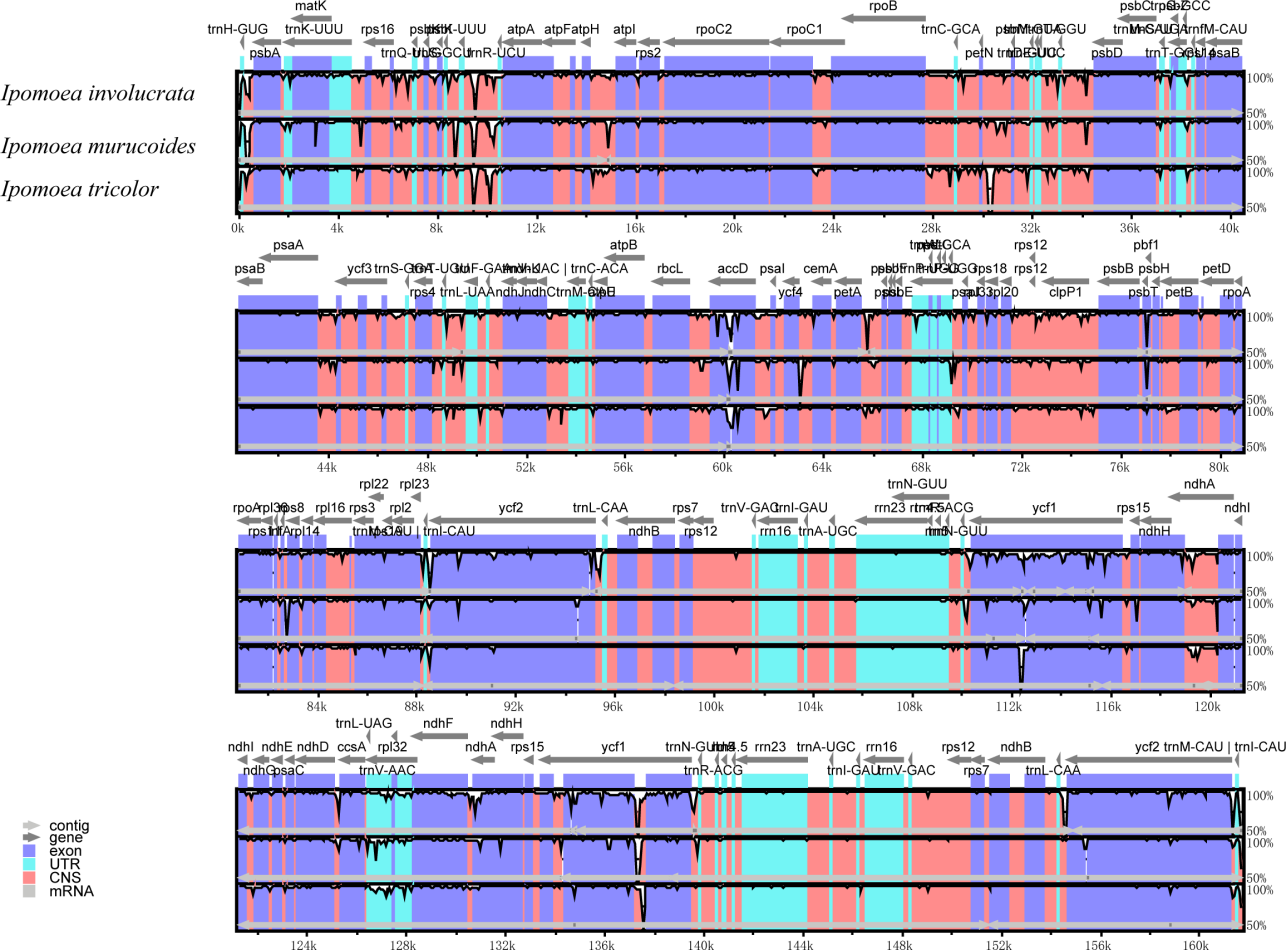
The sequence diversity of the chloroplast genomes of three *Ipomoea* species. The sequence identity plot shows the diversity along the chloroplast genome of *I. involucrate*, *I. murucoides*, and *I. tricolor* with *I. pes-caprae* as a reference using mVISTA. Gray arrows and thick black lines above the alignment indicate genes with their orientation and the position of the IRs, respectively. The colors indicate the exon, UTR, and conserved non-coding sequences (CNS) and mRNA regions. A cutoff of 70% identity was used for the plots, and the Y-scale represents the identity percentage ranging from 50 to 100%.

We also analyzed their chloroplast genomes differences to explore further the genetic relationship among the *I. involucrata*, *I. murucoides*, and *I. tricolor*. The mVISTA program was used for global alignment to exhibit the variation of the chloroplast genomes in different regions using the genome sequence and annotation of *I. pes-caprae* chloroplast as the reference (**Figure 7**). The gene organization was highly conserved across the four chloroplast genomes, with few variation regions, consistent with previous studies (Nguyen et al., 2021). The results also exhibited that the divergences in LSC and SSC regions were higher than in IR regions. Besides, the sequences in the coding regions tended to be more conserved, whereas most of the variations detected were found in conserved non-coding sequences (NCS). The sequences of exons had almost 100% similarity throughout the four taxa. Among the coding genes, the highly disparate sequences are the regions harbouring *rpl2*, *ycf2*, *ndhK*, *ndhD*, and *ycf1* genes.

**Figure 8:**
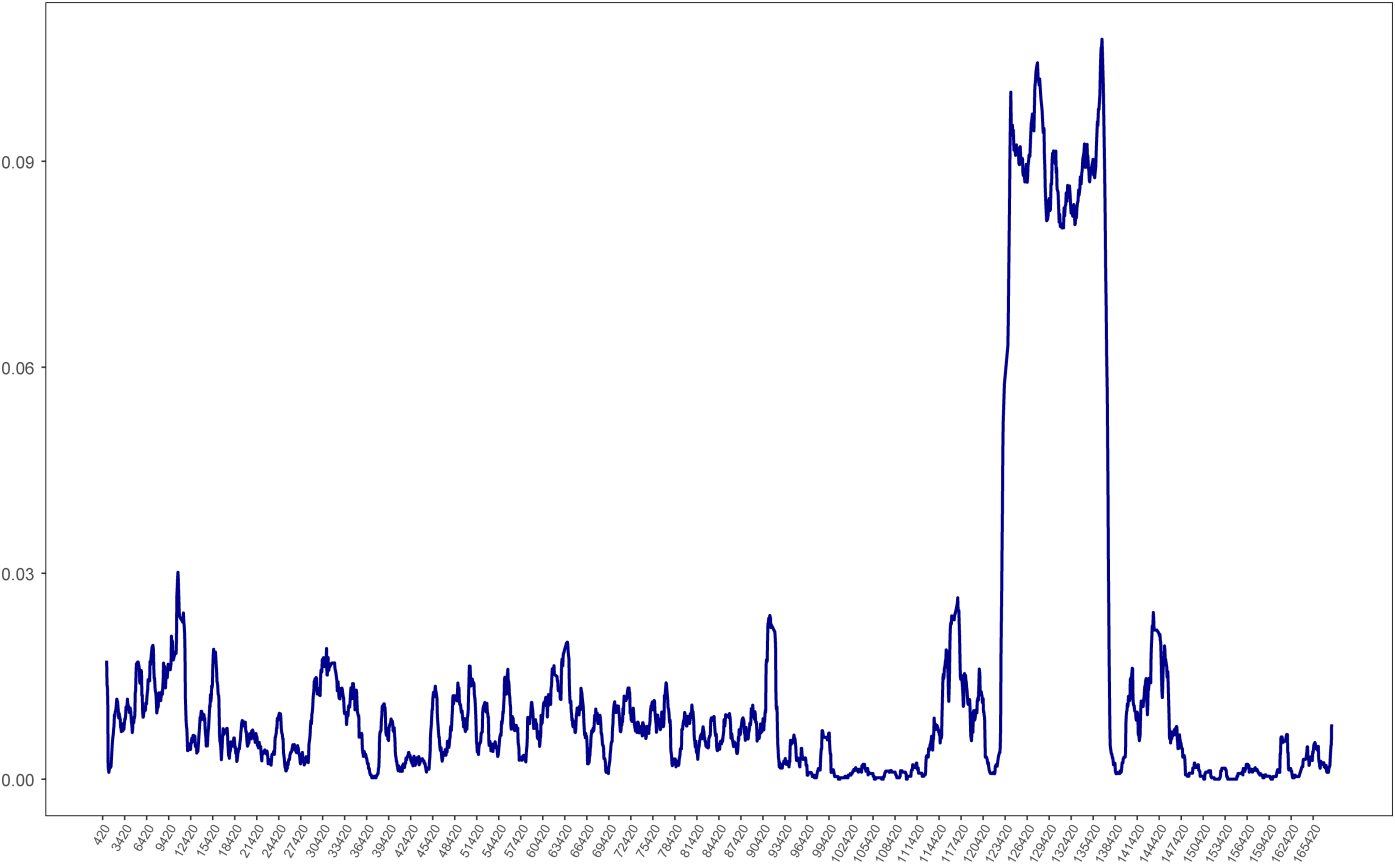
Chloroplast genome comparative analysis between *I. pes-caprae* and other 11 *Ipomoea* species. Sliding window plots of nucleotide diversity (π) across the complete cp genomes of *I pes-caprae* and other 11 *Ipomoea species* (window length: 600 bp, step size: 100 bp). Y-axes: nucleotide diversity (π) of each window; X-axes: the position of the midpoint of a window.

To visualize the overall sequence divergence of the 12 *Ipomoea* species, the *pi* values of the chloroplast genome sequence were calculated with a slide window length of 600 bp and a step size of 100 bp. The sliding window *pi* plots showed that the average *pi* value of the LSC (*Pi* = 0.008101) and SSC (*Pi* = 0.054394) regions was much higher than that in the IR (*Pi* = 0.001942) regions, which showed that LSC and SSC regions contained the most of the variation (**Figure 8**). The plots also showed that the SSC region is hypervariable. Further investigation revealed that most of the SSC region of *I. pes-caprae* wa*s* much shorter than other *Ipomoea* species, indicating an invasion of IRs to this region. These regions with higher *pi* values are more variables that might experience rapid nucleotide substitution and could be used to develop molecular markers for identification and phylogenetic analysis (Lyu et al., 2020).

## Discussion

The chloroplasts are the semi-autonomous organelles in green plants, algae, and cyanobacteria. The main function of chloroplast is to carry out photosynthesis converting the light energy to chemical energy, which is critical for the autotrophic characteristics of those species (Howe et al., 2003; Daniell et al., 2021). Convolvulaceae is a family of about 60 genera and more than 1,650 species of mostly herbaceous vines, trees, shrubs, herbs, and the sweet potato and a few other food tubers (van Ooststroom and Hoogland, 1953; Stefanović et al., 2003). In this family, *Ipomoea* is the largest genus, with over 600 large diversity species with common names such as morning glory, water convolvulus or kangkung, sweet potato, bindweed, and moonflower (Gunn, 1972). The *Ipomoea* species includes food species. For example, *I. batatas* and *I. aquatica* are important food sources for humans and animals (Meira et al., 2012; Mohanraj and Sivasankar, 2014). Some other *Ipomoea* species, for example, *I. Carnea*, *I. quamoclit*, *I. jalapa*, and *I. simulans*, are renowned for their properties in folk medicine and herbalism (Sharma and Bachheti, 2013; Paul and Sinha, 2016); *Cuscuta* is another typical genus in the *Convolvulaceae* family, well-known for their parasitism characteristics, It is composed of over 201 species of yellow, orange, or red (rarely green) parasitic plants, comely found throughout the temperate and tropical regions of the world, with the greatest species diversity in subtropical and tropical regions (Machado and Zetsche, 1990). Since the *Cuscuta* plants could not conduct photosynthesis and had to uptake nutrition from the host plants, they became an ideal model system for studying the communication between plants recently (Hettenhausen et al., 2017; Shahid et al., 2018; Vogel et al., 2018; Sun et al., 2018; Zhuang et al., 2018; Li et al., 2020). In this study, the chloroplast genome of *I. pes-caprae* was assembled and annotated, and comparative analyses of the chloroplast genome of *Convolvulaceae* were conducted.

The chloroplast genome phylogenetic trees were usually used to describe the taxonomical and evolutionary relationships among the plant species. The chloroplast genome phylogeny revealed that 2 *Cuscuta* species (*C. exaltata* and *C. reflexa*) were closely related to the *Ipomoea* species (**Figure S1**), which conflicted with their taxonomic positions. While constructing phylogenetic analysis, we found that the length of their chloroplast genome severely influenced the positions of specific species on the phylogenetic tree. The chloroplasts with a similar genome size tend to have a closer phylogenetic relationship. Since the chloroplast genomes of land plants are conserved in gene order and organization, while the order of four regions might be different for using different assembly and annotation strategies, we reconstructed a phylogenetic tree with a specific order (LSC-IRb-SSC-IRa). The new phylogenetic tree was slightly different from the previous one. However, two *Cuscuta* species, *C. exaltata*, and *C. reflexa, were* still clustered close to *Ipomoea* species. The coding sequences are much more conserved than the non-coding regions during evolution. Therefore, the conservative single-copy genes of the chloroplast genome were extracted and used to construct the phylogenetic tree. As shown in **Figure 5**, *C. exaltata* and *C. reflexa* were clustered together with other *Cuscuta* species, and the phylogenetic relationships of the investigated species highly correspond to their taxonomic relationships. These analyses show that phylogenetic trees constructed based on the conservative single-copy gene sequences are more credible than the complete genome.

The diversity along the chloroplast genome was investigated through *pi* plotting. The results showed that SSC regions of the *Ipomoea* chloroplast were significantly diverse compared to the other areas. Since there are two orientations of the SSC in plant chloroplast genomes (Cheng et al., 2020), which will interfere with calculating the *pi* value, we extensively checked the SSC orientation of the 12 *Ipomoea* chloroplast genomes. The alignments map showed that the SSC orientation of *I. pes-caprae* chloroplast was opposite from these of other *Ipomoea* species (**Supplementary Figure S2**). Therefore, we reversed the SSC region of *I. pes-caprae* and recalculated the *pi* value of the 12 *Ipomoea* chloroplast genomes. The *pi* plots still showed a significant peak in the SSC regions. This analysis indicates that high diversity in the SSC region is indeed excited in the *Ipomoea* species. The mVISTA analysis showed the sequence variation among three *Ipomoea* species, *I. involucrata*, *I. murucoides*, and *I. tricolor* (**Figure 7**). There were many small blocks with low identity in the SSC regions, especially in genes *ycf1* and *rpl32*. We can conclude that the SSC regions are highly diverse, attributed to the diversity of the genes in these regions, such as *ycf1* and *rpl32*.

The comparative analysis of the chloroplast genomes of *Ipomoea* and *Cuscuta* species showed that *Cuscuta* species belong to parasitic plants, and their chloroplast genomes were shorter than their close relative *Ipomoea* species. Therefore the missing genes of *Cuscuta* species could provide clues to deciphering the evolutionary history of the parasite plants. In the *Cuscuta* species, the CDS number is ranged from 31 to 67, with an average of 54.85. However, the CDS number of *Ipomoea* species is varied from 85 to 87, with an average of 85.4. There were 16 CDSs (*pbf1*, *ndhB*, *ndhH*, *ndhE*, *infA*, *ndhJ*, *ndhG*, *ndhC*, *ndhA*, *ndhF*, *ycf15*, *rpl23*, *rps16*, *ndhK*, *ndhI*, and *ndhD*) existing in *Ipomoea species* but not existing in *Cuscuta* species. These CDSs coding genes are mainly involved in photosynthesis. That might explain the loss of the photosynthesis function of *Cuscuta* species during evolution (Funk et al., 2007).

## Data availability statement

The data presented in the study are deposited in the NCBI repository, accession number MZ557416.

## Author contributions

YC, YQ, and WL conceived and designed the research. YW, JX, BH, CD, JS, ZL, KY, and FD performed the experiments. LW and MA helped with a critical discussion on the work. YC and YW wrote the paper. MA, WL, and YQ revised the paper All authors contributed to the article and approved the submitted version.
