# Supplementary material for: Assembly, annotation, and comparative analysis of Ipomoea chloroplast genomes provide insights into the parasitic characteristics of Cuscuta species
Source: Front Plant Sci. 2023 Jan 17;13:1074697. doi: 10.3389/fpls.2022.1074697 (PMC9887335; doi:10.3389/fpls.2022.1074697)
Supplement: Supplementary file 1 [file DataSheet_1.pdf]

Supplementary Table 1. Information of reported chloroplast genomes in the *Convolvulaceae* family

organism	BioProject	Size(Mb)	GC%	Type	Replicons	CDS
Amborella trichopoda	PRJNA12242	0.162686	38.337	chloroplast	NC_005086.1	84
Arabidopsis thaliana	PRJNA116	0.154478	36.2938	chloroplast	NC_000932.1	85
Cuscuta australis	PRJNA608384	0.085263	37.8206	chloroplast	NC_045885.1	61
Cuscuta bonafortunae	PRJNA553874	0.082346	37.2744	chloroplast	NC_043871.1	59
Cuscuta campestris	PRJNA700230	0.086727	37.7218	chloroplast	NC_052920.1	60
Cuscuta carnosa	PRJNA553976	0.081577	37.8305	chloroplast	NC_043868.1	58
Cuscuta chapalana	PRJNA553900	0.084607	37.5548	chloroplast	NC_043870.1	61
Cuscuta costaricensis	PRJNA550927	0.086691	37.1446	chloroplast	NC_042956.1	60
Cuscuta exaltata	PRJNA27795	0.125373	38.1159	chloroplast	NC_009963.1	67
Cuscuta gronovii	PRJNA20671	0.086744	37.7167	chloroplast	NC_009765.1	62
Cuscuta mexicana	PRJNA553975	0.083526	37.5069	chloroplast	NC_043869.1	57
Cuscuta pentagona	PRJNA504171	0.08638	37.8699	chloroplast	NC_039759.1	61
Cuscuta reflexa	PRJNA20675	0.121521	38.2206	chloroplast	NC_009766.1	69
Ipomoea batatas	NA	0.161466	37.5732	chloroplast	KF242475.1	85
Ipomoea hederifolia	NA	0.161383	37.5201	chloroplast	KF242484.1	85
Ipomoea involucrata	NA	0.160531	37.4638	chloroplast	KF242485.1	85
Ipomoea minutiflora	NA	0.16119	37.5706	chloroplast	KF242498.1	85
Ipomoea murucoides	NA	0.160072	37.6368	chloroplast	KF242486.1	85
Ipomoea nil	PRJNA344313	0.161897	37.4652	chloroplast	NC_031159.1	87
Ipomoea obscura	NA	0.16125	37.5256	chloroplast	KF242499.1	85
Ipomoea pes-caprae	NA	0.161667	37.5649	chloroplast	Ipomoea-pe	94
Ipomoea purpurea	PRJNA20803	0.162046	37.4826	chloroplast	NC_009808.1	85
Ipomoea tricolor	NA	0.160776	37.5759	chloroplast	KF242495.1	85
Ipomoea trifida	PRJNA387787	0.161133	37.6192	chloroplast	NC_034670.1	86
Ipomoea triloba	PRJNA479198	0.16175	37.5134	chloroplast	NC_037913.1	85
Oryza sativa Japonica Group	PRJNA122	0.134525	38.9861	chloroplast	NC_001320.1	108
Pinus thunbergii	PRJNA12228	0.119707	38.4957	chloroplast	NC_001631.1	123

Supplementary Table 2. The statistics of chloroplast genome reads in *I. pes-caprae.*

Feature	Value
**number of seq**	115700
**min length (bp)**	488
**max length (bp)**	43051
**total size (bp)**	1901428671
**N90 (bp)**	12913
**N80 (bp)**	13970
**N70 (bp)**	14974
**N60 (bp)**	15879
**N50 (bp)**	16859
**Average length (bp)**	16434

Supplementary Table 3. Summary of the chloroplast genome of *I. pes-caprae.*

Genome features	*Ipomoea pes-caprae*
**Genome size (bp)**	161667
**LSC size (bp)**	88210
**SSC size (bp)**	12117
**IR size (bp)**	30670
**GC content (%)**	37.56
**No. of genes**	136
**No. of PCGs**	86
**No. of tRNA**	42
**No. of rRNA**	8

Supplementary Table 4. SSR in the chloroplast genome of *I. pes-caprae.*

SSR nr.	SSR type	SSR	Size	Start	End	Annotation
1	p1	(A)10	10	6869	6878	IGS(rps16,trnQ-UUG)
2	p1	(A)12	12	8232	8243	IGS(psbI,trnS-GCU)
3	p1	(A)10	10	8495	8504	IGS(trnS-GCU,trnK-UUU)
4	p1	(T)10	10	10237	10246	IGS(trnK-UUU,trnR-UCU)
5	p1	(A)11	11	10384	10394	IGS(trnK-UUU,trnR-UCU)
6	p1	(T)10	10	12931	12940	atpF(intron)
7	p1	(T)10	10	13590	13599	IGS(atpF,atpH)
8	p1	(A)10	10	16104	16113	IGS(atpI,rps2)
9	c	(A)10gaaacgaggaattttgaatttc(A)10	42	16932	16973	IGS(rps2,rpoC2)
10	p1	(T)13	13	19154	19166	rpoC2
11	p1	(A)10	10	23190	23199	rpoC1(intron)
12	p1	(T)10	10	26956	26965	rpoB
13	p1	(A)10	10	28507	28516	IGS(rpoB,trnC-GCA)
14	p1	(T)11	11	30448	30458	IGS(petN,psbM)
15	p1	(T)10	10	31529	31538	IGS(psbM,trnD-GUC)
16	c	(A)11g(A)11	23	31821	31843	IGS(psbM,trnD-GUC)
17	p1	(T)13	13	37025	37037	IGS(psbC,trnS-UGA)
18	p1	(A)11	11	38243	38253	IGS(trnT-GGU,trnM-CAU)
19	p1	(A)13	13	48468	48480	IGS(rps4,trnT-UGU)
20	p1	(T)13	13	49031	49043	IGS(trnT-UGU,trnL-UAA)
21	p1	(A)10	10	52869	52878	IGS(ndhC,trnV-UAC)
22	p1	(T)10	10	54684	54693	IGS(trnM-CAU,atpE)
23	p1	(T)10	10	56667	56676	atpB
24	p2	(TA)7	14	64393	64406	IGS(cemA,petA)
25	p1	(A)13	13	65917	65929	IGS(petA,psbJ)
26	p1	(A)10	10	71810	71819	IGS(rpl20,rps12)
27	p1	(T)10	10	72321	72330	IGS(rpl20,rps12)
28	p1	(A)10	10	73369	73378	IGS(rps12,psbB)
29	p1	(A)10	10	85164	85173	rpl16(intron)
30	p1	(A)12	12	111285	111296	ycf1
31	p1	(A)10	10	111696	111705	ycf1
32	p1	(A)13	13	112886	112898	ycf1
33	p1	(T)13	13	136980	136992	ycf1
34	p1	(T)10	10	138173	138182	ycf1
35	p1	(T)12	12	138582	138593	ycf1

Supplementary Table 5. The long repeats in the chloroplast genome of *I. pes-caprae.*

Start	End	Repeat Type	Start	End	maximum allowed distance	minimum repeat size	E-value
30670	88210	P	30670	130997	0	0	0.00E+00
234	60037	F	234	60061	0	9	6.40E-131
242	112368	F	242	112413	-3	9	2.70E-128
242	112368	P	242	137222	-3	9	2.70E-128
242	112413	P	242	137267	-3	9	2.70E-128
242	137222	F	242	137267	-3	9	2.70E-128
232	112407	F	232	112452	-3	8	5.60E-122
232	112407	P	232	137193	-3	8	5.60E-122
232	112452	P	232	137238	-3	8	5.60E-122
232	137193	F	232	137238	-3	8	5.60E-122
210	60037	F	210	60085	0	2	7.10E-116
212	112427	F	212	112472	-2	3	4.20E-112
212	112427	P	212	137193	-2	3	4.20E-112
212	112472	P	212	137238	-2	3	4.20E-112
186	60037	F	186	60109	0	7	6.40E-102
175	112476	F	175	112521	-3	7	6.00E-88
175	112476	P	175	137181	-3	7	6.00E-88
175	112521	P	175	137226	-3	7	6.00E-88
175	137181	F	175	137226	-3	7	6.00E-88
162	60037	F	162	60133	0	2	1.50E-87
163	112431	F	163	112521	-2	6	3.90E-83
163	112431	P	163	137193	-2	6	3.90E-83
163	112521	P	163	137283	-2	6	3.90E-83
163	137193	F	163	137283	-2	6	3.90E-83
158	112407	F	158	112497	-3	9	5.90E-78
158	112407	P	158	137222	-3	9	5.90E-78
158	112497	P	158	137312	-3	9	5.90E-78
158	137222	F	158	137312	-3	9	5.90E-78
154	115549	F	154	115573	-2	1	4.90E-77
154	115549	P	154	134150	-2	1	4.90E-77
154	115573	P	154	134174	-2	1	4.90E-77
154	134150	F	154	134174	-2	1	4.90E-77
138	60037	F	138	60157	0	6	5.00E-74
130	112476	F	130	112566	-3	3	8.30E-61
130	112476	P	130	137181	-3	3	8.30E-61
130	112566	P	130	137271	-3	3	8.30E-61
130	137181	F	130	137271	-3	3	8.30E-61
119	115584	F	119	115608	-1	5	9.40E-59
119	115584	P	119	134150	-1	5	9.40E-59
119	115608	P	119	134174	-1	5	9.40E-59
114	60037	F	114	60181	0	1	7.00E-58

113	115590	F	113	115614	0	6	8.20E-58
113	115590	P	113	134150	0	6	8.20E-58
113	115614	P	113	134174	0	6	8.20E-58
118	112431	F	118	112566	-2	4	1.40E-56
118	112431	P	118	137193	-2	4	1.40E-56
118	112566	P	118	137328	-2	4	1.40E-56
118	137193	F	118	137328	-2	4	1.40E-56
119	115560	F	119	115608	-3	1	2.30E-54
119	115560	P	119	134150	-3	1	2.30E-54

Supplementary Table 6. Frequency of 20 amino acids in the chloroplast genome of *I. pes-caprae*.

Amino acid	Count	Percentage
**Leu**	5708	0.105921
**Ser**	4961	0.09206
**Ile**	4470	0.082948
**Phe**	3892	0.072223
**Lys**	3530	0.065505
**Arg**	3486	0.064689
**TER**	2975	0.055206
**Pro**	2595	0.048155
**Val**	2564	0.047579
**Asn**	2478	0.045983
**Gly**	2315	0.042959
**Thr**	2287	0.042439
**Glu**	2053	0.038097
**Tyr**	1976	0.036668
**Ala**	1762	0.032697
**Gln**	1484	0.027538
**Asp**	1416	0.026276
**His**	1195	0.022175
**Cys**	1127	0.020913
**Met**	902	0.016738
**Trp**	713	0.013231

Supplementary Table 7. RSCU and counts of codons in the chloroplast genome of *I. pes-caprae*.

Amino acid	Codon	Count	RSCU	Amino acid	Codon	Count	RSCU
Phe	UUU	2373	1.22	Tyr	UAU	1370	1.39
Phe	UUC	1519	0.78	Tyr	UAC	606	0.61
Leu	UUA	1177	1.24	TER	UAA	1034	1.04
Leu	UUG	1192	1.25	TER	UAG	774	0.78
Leu	CUU	1162	1.22	His	CAU	826	1.38
Leu	CUC	701	0.74	His	CAC	369	0.62
Leu	CUA	960	1.01	Gln	CAA	1014	1.37
Leu	CUG	516	0.54	Gln	CAG	470	0.63
Ile	AUU	1873	1.26	Asn	AAU	1713	1.38
Ile	AUC	1069	0.72	Asn	AAC	765	0.62
Ile	AUA	1528	1.03	Lys	AAA	2419	1.37
Met	AUG	902	1	Lys	AAG	1111	0.63
Val	GUU	936	1.46	Asp	GAU	991	1.4
Val	GUC	438	0.68	Asp	GAC	425	0.6
Val	GUA	743	1.16	Glu	GAA	1417	1.38
Val	GUG	447	0.7	Glu	GAG	636	0.62
Ser	UCU	1326	1.6	Cys	UGU	700	1.24
Ser	UCC	840	1.02	Cys	UGC	427	0.76
Ser	UCA	1127	1.36	TER	UGA	1167	1.18
Ser	UCG	632	0.76	Trp	UGG	713	1
Pro	CCU	705	1.09	Arg	CGU	410	0.71
Pro	CCC	638	0.98	Arg	CGC	255	0.44
Pro	CCA	780	1.2	Arg	CGA	657	1.13
Pro	CCG	472	0.73	Arg	CGG	421	0.72
Thr	ACU	681	1.19	Ser	AGU	633	0.77
Thr	ACC	572	1	Ser	AGC	403	0.49
Thr	ACA	684	1.2	Arg	AGA	1157	1.99
Thr	ACG	350	0.61	Arg	AGG	586	1.01
Ala	GCU	565	1.28	Gly	GGU	580	1
Ala	GCC	361	0.82	Gly	GGC	347	0.6
Ala	GCA	539	1.22	Gly	GGA	791	1.37
Ala	GCG	297	0.67	Gly	GGG	597	1.03

Supplementary Table 8. Gene types and count in the chloroplast genome of *Ipomoea* and *Cuscuta*

Species	Number of gene type	Gene count
Ipomoea batatas	85	122
Ipomoea hederifolia	85	122
Ipomoea involucrata	85	121
Ipomoea minutiflora	85	122
Ipomoea murucoides	85	122
Ipomoea nil	87	132
Ipomoea obscura	85	122
Ipomoea pes-caprae	87	136
Ipomoea purpurea	85	131
Ipomoea tricolor	85	122
Ipomoea trifida	86	132
Ipomoea triloba	85	131
Cuscuta australis	61	97
Cuscuta exaltata	67	117
Cuscuta reflexa	67	113
Cuscuta chapalana	61	96
Cuscuta gronovii	61	98
Cuscuta pentagona	61	97
Cuscuta campestris	60	96
Cuscuta costaricensis	60	96
Cuscuta bonafortunae	58	96
Cuscuta carnosa	58	91
Cuscuta mexicana	57	92
Cuscuta erosa	33	71
Cuscuta strobilacea	33	74
Cuscuta boldinghii	31	70

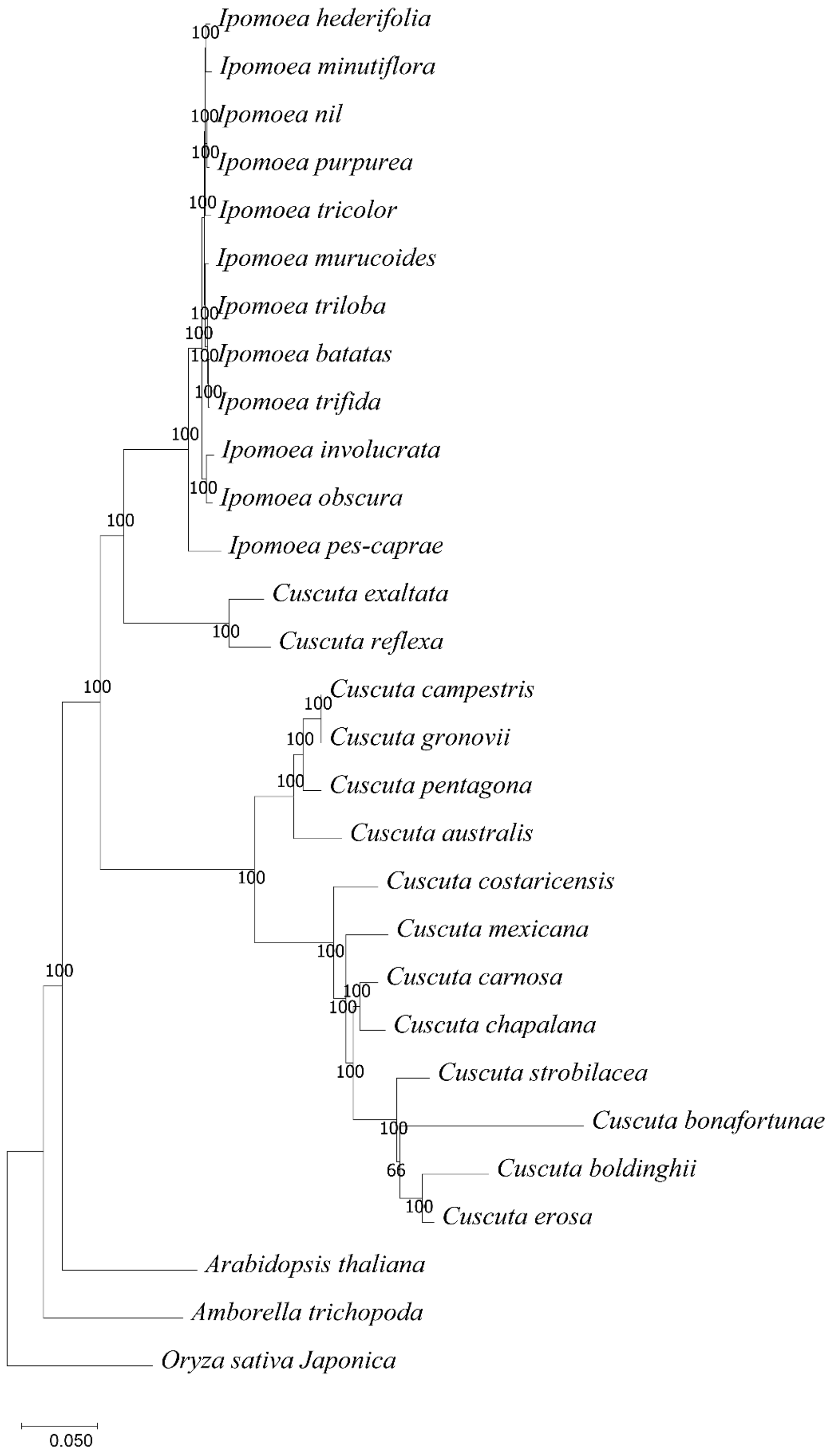

Figure S1. The phylogenetic tree of 29 species was constructed using the complete chloroplast genome sequences using the Maximum likelihood (ML) method.

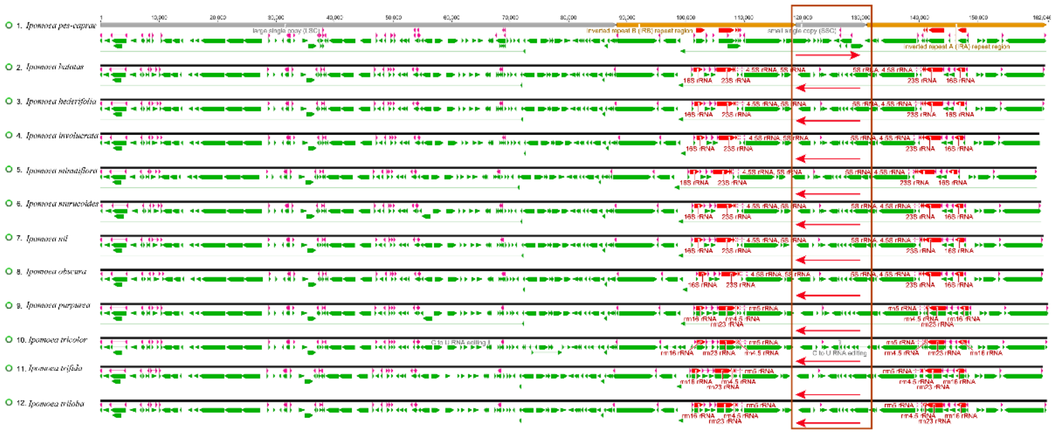

Figure S2. The visualization of the 12 *Ipomoea* chloroplast genomes shows the orientations of SSC regions.
